# Single-Cell RNA Sequencing in Porcine Biology and Production

**DOI:** 10.3390/genes17070731

**Published:** 2026-06-24

**Authors:** Xia Zhang, Yunze Deng, Xiaojing Hu, Hailong Huo, Jinlong Huo

**Affiliations:** 1 Department of Biological and Food Engineering, Lyuliang University, Lvliang 033001, China; xiazhang1425@163.com (X.Z.);; 2 Yunnan Open University, Kunming 650223, China; 3 College of Animal Science and Technology, Yunnan Agricultural University, Kunming 650201, China

**Keywords:** single-cell RNA sequencing, porcine, cellular heterogeneity, reproduction, livestock production

## Abstract

Single-cell RNA sequencing (scRNA-seq) has emerged as a transformative technology for resolving cellular heterogeneity and deciphering gene regulatory networks in complex tissues. Despite challenges such as incomplete genome annotation, technical variability across platforms, and limitations in robust cell-type annotation, scRNA-seq has substantially advanced our understanding of the developmental processes, physiological regulation, and disease responses in pigs, an economically and biomedically important species, thereby providing insights into traits of agricultural and translational relevance. By profiling transcriptomes at the single-cell resolution, scRNA-seq enables the identification of rare cell populations, dynamic cellular states, and lineage trajectories that are critical for reproduction, growth, immunity, and metabolic homeostasis. Recent porcine scRNA-seq studies have generated high-resolution cellular atlases spanning embryos, reproductive organs, immune tissues, skeletal muscle, and the gastrointestinal tract, revealing cell-type-specific regulatory mechanisms associated with reproductive performance, muscle accretion, adipogenesis, immune competence, and intestinal functionality. This review summarizes the fundamental principles and analytical strategies of scRNA-seq, highlights its major applications in porcine biology and production, and discusses current challenges as well as future perspectives for integrating single-cell technologies into livestock science.

## 1. Introduction

Pigs are among the most economically important livestock worldwide, as pork accounts for a substantial proportion of global meat consumption, and production has increased dramatically to meet growing demand [1]. Beyond their agricultural value, pigs are also widely used as biomedical models due to their physiological and anatomical similarities to humans [2,3]. Enhancing reproductive efficiency, growth performance, immune competence, and disease resistance remains a central goal of modern pig breeding programs [4,5]. Achieving these objectives requires a detailed understanding of the cellular and molecular mechanisms that govern porcine development, physiology, and adaptation. Conventional bulk transcriptomic studies have advanced knowledge of gene regulation in pigs [6]; however, these approaches average signals across heterogeneous cell populations, masking cell-type-specific transcriptional programs and rare but functionally important cell subsets. Critical biological processes, including embryogenesis, spermatogenesis, immune responses, and intestinal homeostasis, are orchestrated by highly dynamic and heterogeneous cell populations [7,8], highlighting the need for technologies capable of resolving gene expression at the single-cell resolution [9].

Single-cell RNA sequencing (scRNA-seq) has emerged as a transformative tool, enabling unbiased, high-resolution profiling of transcriptomes at the level of individual cells, resolving cellular heterogeneity, and revealing rare cell types that determine tissue function and disease prognosis [10]. Despite the limitations of this technology, such as cell dissociation bias, batch effects, high shedding rates, enormous computational requirements, and incomplete pig genome annotation, it can still achieve significant results by capturing cellular heterogeneity, reconstructing developmental trajectories, and identifying novel cell types and states; scRNA-seq provides unprecedented insight into complex biological systems [11]. In pigs, scRNA-seq has facilitated the construction of high-resolution cellular atlases across multiple tissues and developmental stages, advancing our understanding of embryogenesis [12], reproduction [13], immunity [14], growth and muscle development [15], and gut physiology [16]. These studies not only deepen our knowledge of porcine biology but also offer valuable resources for precision breeding and disease control. This review summarizes the principles and analytical frameworks of scRNA-seq, highlights its applications in porcine biology and production, and discusses challenges and future directions for integrating single-cell approaches into swine production.

## 2. Overview of Single-Cell RNA Sequencing in Porcine Research

Single-cell RNA sequencing (scRNA-seq) has emerged as a transformative technology for dissecting cellular heterogeneity and uncovering cell-type-specific gene expression programs in complex tissues. Unlike bulk RNA sequencing, which averages transcriptomic signals across mixed cell populations, scRNA-seq profiles individual cells, thereby enabling high-resolution and quantitative characterization of rare cell types, dynamic cellular states, and developmental trajectories [17,18].

In livestock species such as pigs, this high-resolution approach provides unique opportunities to investigate the biological processes directly relevant to reproduction, growth, immunity, and overall health, which collectively underpin production efficiency and genetic improvement [19,20]. Recent advances, including expanded porcine genomic resources and continually decreasing sequencing costs, have facilitated the application of scRNA-seq to embryos, reproductive organs, immune tissues, and metabolically active organs [21]. Collectively, these studies are progressively constructing a comprehensive single-cell atlas of porcine tissues, offering a valuable framework for understanding complex biological systems and translating molecular insights into precision breeding and health management strategies.

### 2.1. Basic Principles of Single-Cell RNA Sequencing

**Figure 1:**
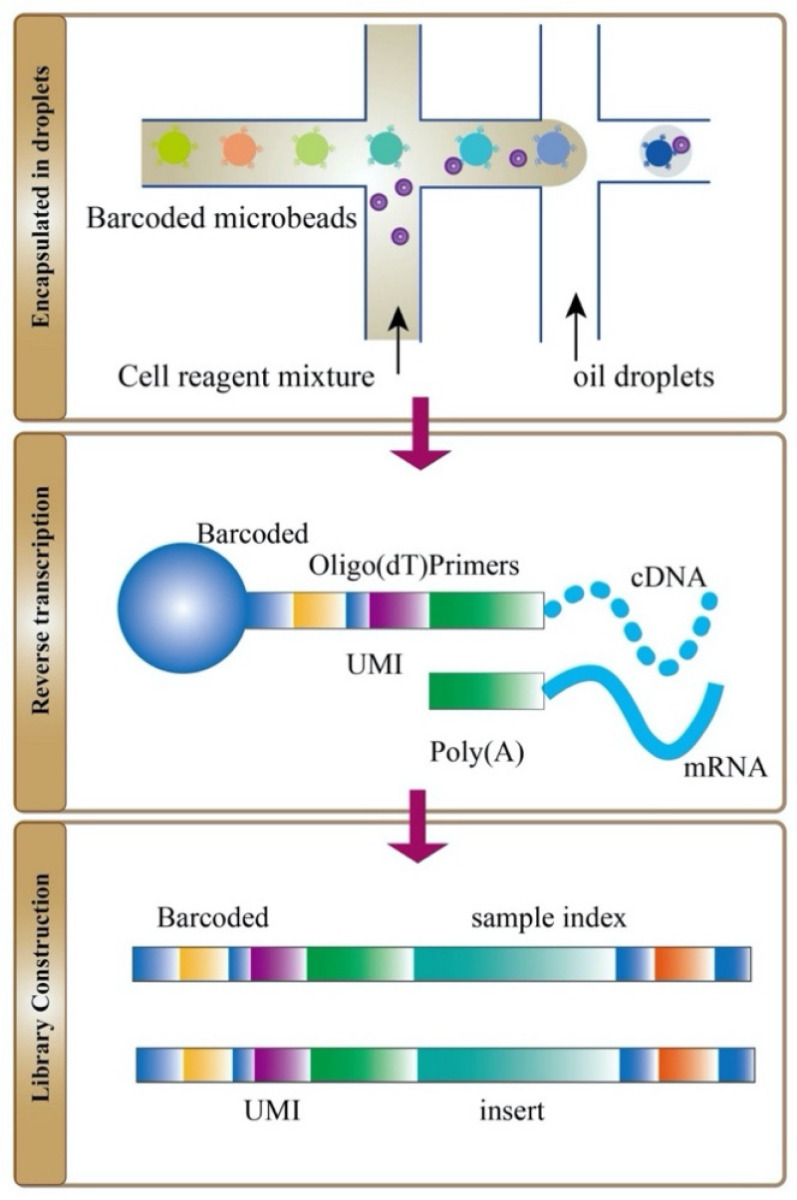
Overview of the scRNA-seq workflow. Tissues are dissociated into single-cell suspensions, followed by cell capture, barcoding, and UMI labeling. After reverse transcription and library construction, sequencing reads are generated, demultiplexed, and mapped to a reference genome to produce a gene-by-cell expression matrix.

The core principle of single-cell RNA sequencing (scRNA-seq) is the isolation and transcriptomic profiling of individual cells, which allows researchers to dissect cellular heterogeneity and identify rare or transient cell populations that are often masked in bulk RNA sequencing. In a typical workflow (Figure 1), tissues are enzymatically or mechanically dissociated into single-cell suspensions [22]. The quality of tissue dissociation is critical, as it affects cell viability, the recovery of diverse cell types, and the fidelity of downstream analyses [23,24]. Therefore, optimized and tissue-specific dissociation protocols are essential for preserving cell integrity and minimizing transcriptional artifacts.

Once single-cell suspensions are prepared, individual cells are captured using techniques such as microfluidic devices, droplet-based systems, or microwell arrays [25]. Each cell is labeled with a unique barcode, and each transcript is tagged with a unique molecular identifier (UMI) [26]. This dual labeling ensures accurate quantification of gene expression while controlling for PCR amplification bias [27,28]. Reverse transcription and library construction convert these barcoded RNAs into sequencing-ready libraries, which are then subjected to high-throughput sequencing. After sequencing, reads are demultiplexed based on cell-specific barcodes and mapped to a reference genome, producing a gene-by-cell expression matrix [29].

**Figure 2:**
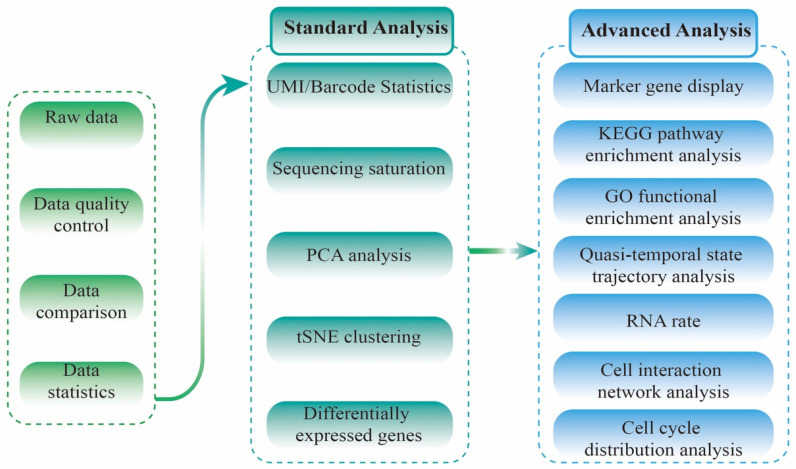
Workflow of downstream scRNA-seq data analysis. Following sequencing, the data underwent quality control, normalization, dimensionality reduction, clustering, and marker gene identification to define distinct cell populations and infer their functional states. Advanced analyses, such as trajectory inference and pseudotime reconstruction, model dynamic biological processes and lineage relationships.

Downstream bioinformatic analyses (Figure 2) typically include quality control, normalization, dimensionality reduction, clustering, and marker gene identification, which collectively allow the definition of distinct cell populations and inference of their functional states [30,31,32]. More advanced analyses, such as trajectory inference and pseudotime reconstruction, enable modeling of dynamic biological processes and lineage relationships [33].

In the context of porcine biology, scRNA-seq has been instrumental in identifying germ cell subtypes, somatic support cells, immune cell populations, and metabolically specialized cell subsets that are often obscured in bulk analyses. Importantly, scRNA-seq can reconstruct developmental and physiological trajectories, revealing processes such as embryonic lineage specification [12], spermatogenic progression [34], immune cell differentiation [14], and intestinal epithelial renewal [35]. By capturing both static cell types and dynamic cellular states at a high resolution, scRNA-seq provides a powerful framework for investigating mechanisms that directly impact growth, reproduction, immunity, and overall productivity in pigs.

Additionally, integration with complementary single-cell multi-omics approaches—such as chromatin accessibility profiling (scATAC-seq) [36], protein expression analysis (CITE-seq) [37], and spatial transcriptomics [38]—further enhances our ability to connect transcriptional states with epigenetic regulation, protein activity, and tissue architecture, offering a more holistic view of cellular function in porcine tissues.

### 2.2. Single-Cell RNA Sequencing Platforms Used in Porcine Studies

Single-cell RNA sequencing (scRNA-seq) platforms used in porcine research can be broadly classified into plate-based [39,40], droplet-based [41], and multimodal systems, each offering distinct advantages and limitations depending on the research objective and tissue type. Plate-based approaches, such as Smart-seq2 [42,43,44] and SMARTer ICELL8 [45,46], provide high sensitivity and full-length transcript coverage, making them ideal for low-input samples like early embryos and rare cell populations. However, their low throughput and high cost limit large-scale applications. Droplet-based platforms, most notably 10× Genomics Chromium [43], as well as Drop-seq and inDrop [47,48], are widely used for high-throughput profiling of heterogeneous cell populations, enabling comprehensive cellular atlas construction across porcine tissues including the testis, intestine, liver, skeletal muscle, and immune organs. These systems encapsulate single cells into nanoliter-sized droplets together with barcoded beads, thereby allowing massively parallel transcriptome profiling at a relatively low cost per cell.

Emerging domestic platforms, such as Singleron GEXSCOPE [49,50], as well as micro-well based systems like BD Rhapsody [51], offer cost-effective, high-sensitivity alternatives suitable for large-scale or rare-cell studies. Compared with conventional droplet-based platforms, some of these systems provide flexible experimental designs and improved capture efficiency for specific sample types. Multimodal technologies, including CITE-seq/REAP-seq [52] and 10× Multiome [53], integrate transcriptomic, epigenomic, and proteomic information, providing richer insights into cell states, developmental trajectories, and regulatory networks. The choice of platform in pigs depends on tissue complexity, cell abundance, and research goals. Plate-based methods are optimal for high-resolution, full-length transcript studies, whereas droplet-based systems excel in profiling large heterogeneous cell populations. Although no scRNA-seq platform is universally superior, 10× Genomics Chromium is currently the most commonly used platform in porcine research because it offers an effective balance between throughput, scalability, and transcriptomic coverage for diverse biological applications [8]. Beyond general technical characteristics, the selection of scRNA-seq platforms in porcine research should consider tissue-specific properties, cell recovery performance, transcript sensitivity, and downstream analytical requirements. Future studies should emphasize a biology-driven framework for platform selection to maximize data quality, improve cross-study comparability, and facilitate integrative analyses across diverse porcine tissues and experimental settings. Table 1 summarizes the main scRNA-seq platforms applied in pigs, highlighting their type, throughput, advantages, limitations, and representative applications.

### 2.3. Data Analysis Strategies for Porcine scRNA-Seq Studies

Most porcine single-cell RNA sequencing (scRNA-seq) studies follow a standardized analytical workflow and typically include quality control, normalization, dimensionality reduction, clustering, and cell-type annotation [54]. These core steps ensure that data are rigorously preprocessed, removing technical noise and correcting for confounding factors such as library size, cell viability differences, and batch effects. Following preprocessing, downstream analyses are implemented according to the specific biological questions being addressed.

One key downstream analysis is trajectory inference, which reconstructs the dynamic progression of cellular states across developmental or physiological continua. This approach is particularly relevant in studies of embryonic development, spermatogenesis, and immune cell differentiation in pigs, where scRNA-seq data enable the reconstruction of lineage hierarchies and the identification of key regulatory genes driving cellular transitions [12,34,35]. Another important analysis is differential gene expression, which helps identify genes that are differentially expressed between distinct cell types or developmental stages. This is critical for understanding growth regulation, immune responses, and tissue development in pigs [14,15,16].

Data integration has become increasingly important, particularly in multi-omics contexts where scRNA-seq datasets are combined with other high-dimensional data types, such as chromatin accessibility profiles, protein abundance measurements, or imaging data. Integration frameworks enable cross-sample comparison, batch correction, and multimodal alignment, thereby improving the robustness and interpretability of biological conclusions. Techniques such as single-cell multi-omics and spatial transcriptomics provide a more comprehensive understanding of cellular function within its native tissue context, offering insights into immune regulation, intestinal health, and muscle growth in pigs [49]. Finally, inference of intercellular communication enables the systematic prediction of ligand–receptor interactions between distinct cell populations. This analysis is particularly valuable in studies of gut physiology, immune interactions, and tissue repair, where coordinated signaling networks play a central regulatory role [14].

The major computational tools commonly used in porcine scRNA-seq studies include Seurat, Scanpy, and Monocle for clustering, dimensionality reduction, and trajectory analysis, while tools like CellPhoneDB and NATMI are used to assess cell–cell communication networks. Collectively, these analytical frameworks facilitate high-resolution characterization of cellular heterogeneity and regulatory programs, providing mechanistic insights into complex biological processes such as embryogenesis, immune regulation, and muscle differentiation in pigs [30,31,32]. Table 2 summarizes the principal analytical strategies and computational tools employed in porcine scRNA-seq studies.

## 3. Applications of Single-Cell RNA Sequencing in Porcine Biology and Production

By resolving cellular heterogeneity and capturing dynamic transcriptional programs across diverse tissues and developmental stages, single-cell transcriptomics has provided unprecedented insights into fundamental biological processes and traits of economic significance in pigs. Table 3 summarizes the representative pig single-cell datasets discussed in this review, including the tissue type, sequencing platform, key findings, and public accession numbers.

### 3.1. Porcine Embryonic Development

**Figure 3:**
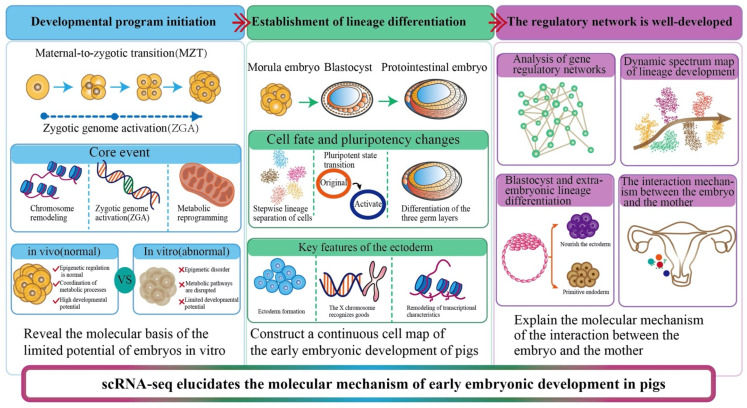
Single-cell transcriptomic landscape of porcine embryonic development. scRNA-seq reveals dynamic transcriptional changes during porcine embryogenesis, including zygotic genome activation, maternal RNA clearance, and metabolic reprogramming at early cleavage stages. Subsequent analyses delineate lineage segregation, pluripotency transitions, and germ-layer formation during peri-implantation development. The single-cell resolution further enables the reconstruction of developmental trajectories and identification of cell-type-specific regulatory networks underlying embryonic competence and early cell fate determination.

Single-cell RNA sequencing (scRNA-seq) has transformed the understanding of porcine early embryogenesis by enabling high-resolution characterization of lineage specification, gene regulatory networks, and dynamic transcriptional programs across successive developmental stages (Figure 3).

At the cleavage stages, scRNA-seq analyses revealed that in vivo-derived porcine embryos undergo tightly coordinated zygotic genome activation (ZGA), chromatin remodeling, and metabolic reprogramming. In contrast, embryos generated in vitro exhibit disrupted epigenetic regulation and perturbed metabolic pathways, highlighting key molecular barriers that limit developmental competence under artificial culture conditions [55]. Complementary studies further delineated the temporal dynamics of maternal RNA clearance and ZGA in embryos derived from in vitro fertilization and parthenogenetic activation, identifying stage-specific regulatory genes and transcriptional divergence between biparental and maternal-only developmental programs [12]. Additionally, single-cell transcriptomic profiling uncovered dynamic expression patterns of long non-coding RNAs during the maternal-to-zygotic transition, implicating lncRNAs as critical regulators of early genome activation in pigs [56].

Beyond the cleavage stages, scRNA-seq has provided comprehensive insights into lineage segregation and pluripotency transitions during peri-implantation development. High-resolution single-cell atlases of pig gastrulation systematically mapped the cell-type composition, lineage trajectories, and transcriptional landscapes of the three germ layers, revealing both conserved and species-specific developmental features compared with human and mouse embryos [57]. Analyses of pre-gastrulating pig embryos further demonstrated stepwise lineage segregation, a transition from native-like states to primed pluripotency states, and X-chromosome inactivation in late epiblast cells, underscoring the pig as an important intermediate model for comparative mammalian developmental biology [58].

The integration of scRNA-seq with functional assays and multi-omics approaches has further enhanced its impact. Guided by single-cell transcriptomic data, stable pig pregastrulation epiblast stem cell lines were successfully established, exhibiting sustained pluripotency, distinct three-dimensional chromatin architecture, and robust genome-editing capacity, ultimately enabling the generation of cloned gene-edited piglets [59]. Moreover, single-cell multi-omics profiling integrating transcriptomic and chromatin accessibility datasets revealed dynamic gene regulatory networks governing embryonic and extra-embryonic lineage differentiation in porcine blastocysts while demonstrating relative transcriptional stability of the epiblast during peri-implantation development [60]. Finally, integrative analyses combining single-cell transcriptomics with uterine fluid proteomics uncovered complex embryo–maternal signaling interactions, identifying functionally specialized trophectoderm subtypes and key ligand–receptor pathways that mediate blastocyst–maternal communication [61].

Collectively, these studies demonstrate that scRNA-seq has advanced porcine embryology from descriptive staging toward a mechanistic understanding of lineage specification, epigenetic regulation, and embryo–maternal interactions. These insights identify cell-type-specific regulatory pathways associated with embryonic competence and implantation potential, providing new opportunities to improve reproductive efficiency in pig production. However, cross-study interpretation remains constrained by differences in embryo sources, sequencing platforms, sample sizes, and cell-state annotation. Future integration of single-cell approaches with functional validation and embryo evaluation systems may support the development of molecular biomarkers for embryo quality assessment and precision breeding in pigs.

### 3.2. Porcine Reproduction

**Figure 4:**
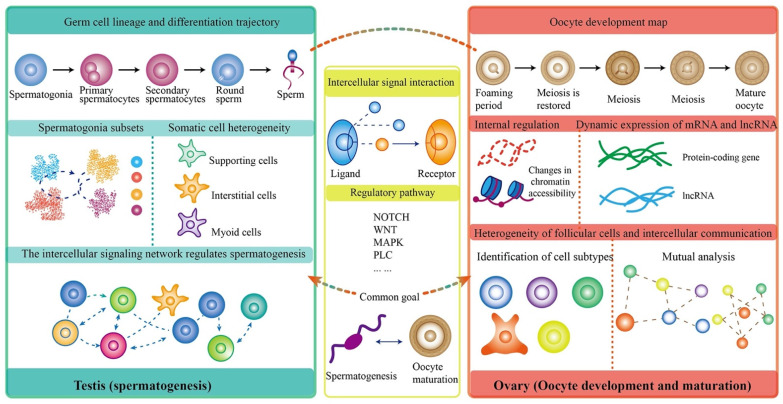
Single-cell transcriptomic profiling of porcine reproduction. scRNA-seq reveals cellular heterogeneity and developmental trajectories in porcine testes and oocytes. In testes, distinct germ cell subpopulations and somatic cell types, along with their regulatory networks and intercellular interactions, are identified during spermatogenesis. In ovaries, single-cell and multi-omics analyses characterize oocyte maturation, follicular cell heterogeneity, and signaling pathways governing folliculogenesis and reproductive competence. These analyses further uncover stage-specific transcriptional programs and candidate molecular regulators associated with gamete development and fertility potential.

Recent advances in single-cell transcriptomics have substantially deepened our understanding of porcine reproductive biology by enabling high-resolution dissection of cellular heterogeneity and developmental dynamics in both testes and oocytes (Figure 4). In adult pig testes, scRNA-seq delineated the dynamic cellular composition and developmental trajectories of germ cells, identifying distinct spermatogonial subpopulations and lineage-specific transcriptional programs. The identification of markers such as *CD99*, *PODXL2*, and *TKTL1* has contributed substantially to the molecular classification of porcine spermatogonia [13,62]. Beyond germ cells, single-cell profiling uncovered substantial somatic cell heterogeneity, including Sertoli, Leydig, and peritubular myoid cells, and revealed complex intercellular signaling networks orchestrating spermatogenesis [63,64]. Cross-species comparisons with human and mouse datasets demonstrated both conserved and species-specific transcriptional programs, particularly during puberty, providing insights into testicular maturation and evolutionary conservation [34]. Breed-specific analyses in indigenous pigs further reconstructed Leydig and myoid cell developmental trajectories, highlighting genetic diversity in reproductive regulation [65]. Large-scale postnatal profiling identified rare cell populations and key transcription factors, such as SOX9 and IRF8, thereby elucidating regulatory networks underpinning testicular development [66].

Complementing testicular studies, single-cell transcriptomics has provided comprehensive insights into porcine oocyte development and maturation. Global transcriptomic profiling revealed dynamic expression patterns of protein-coding genes and long non-coding RNAs (lncRNAs), implicating lncRNAs in meiosis, cellular differentiation, and developmental regulation. Integrative bioinformatic analyses that infer co-expression networks from large-scale repositories further predicted lncRNA-mediated regulatory networks that contribute to oocyte developmental competence [67]. Single-cell multi-omics approaches that employ joint-embedding computational frameworks to integrate transcriptomic and epigenomic datasets uncovered chromatin accessibility landscapes and transcriptional programs that prime germinal vesicle (GV) oocytes for meiotic progression and cytoplasmic maturation by identifying key signaling pathways and candidate regulators associated with oocyte maturation and early embryonic developmental potential [68]. Beyond these intrinsic regulatory mechanisms, environmental perturbation studies demonstrated that toxicant exposure, such as DMBA treatment, disrupts gene expression and lncRNA profiles in oocytes, affecting MAPK, PLC, and nucleotide repair pathways, thereby underscoring the sensitivity of oocyte maturation to oxidative stress and signaling imbalance [69]. Beyond intrinsic regulatory mechanisms, scRNA-seq has characterized cellular heterogeneity and intercellular communication within porcine antral follicles. Distinct granulosa, theca, and germ cell populations were defined, and ligand–receptor analyses revealed dynamic signaling interactions governing folliculogenesis and oocyte maturation [70]. Integration of single-cell and spatial transcriptomics further uncovered cortical–medullary organization, stage-specific gene expression programs, and microenvironmental regulation mediated by the NOTCH, WNT, and extracellular matrix pathways, providing a spatially resolved framework for understanding early oogenesis and its conservation across species [71]. Collectively, these studies establish a comprehensive molecular atlas of porcine gametogenesis, highlighting regulatory networks governing spermatogenesis, oocyte maturation, and follicular microenvironmental interactions. However, to move beyond descriptive atlas-building procedures and confirm the causal roles of these identified candidate regulators, there is a critical need for high-throughput functional validation, such as CRISPR-based screens in porcine cell lines and model systems.

Importantly, these advances extend beyond generating descriptive cellular atlases and provide a conceptual framework for improving reproductive performance in pigs. The identification of cell-type-specific regulatory mechanisms governing spermatogonial maintenance, oocyte competence, follicular communication, and pubertal maturation may facilitate the discovery of biomarkers associated with fertility, litter size, embryo viability, and reproductive longevity. Nevertheless, cross-study interpretation remains constrained by differences in sample origin, developmental stage, sequencing platform, and annotation strategies, underscoring the need for standardized analytical pipelines and validation across genetically diverse pig populations.

### 3.3. Porcine Immune System

**Figure 5:**
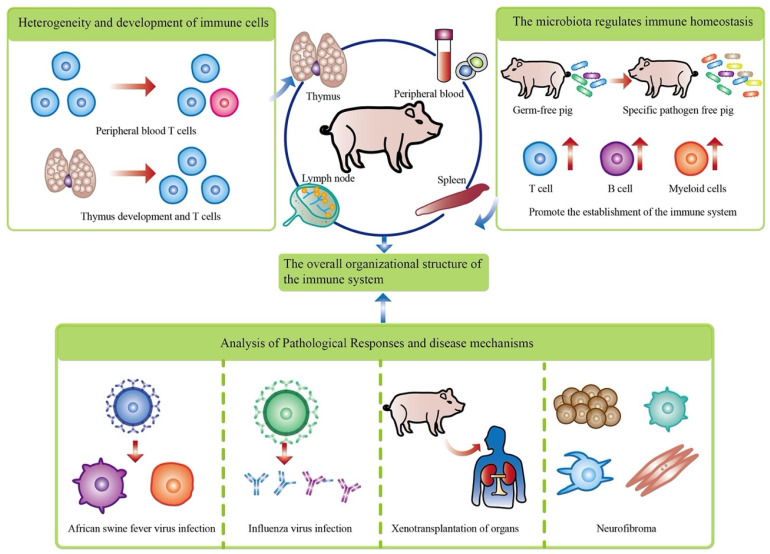
Single-cell transcriptomic landscape of the porcine immune system. scRNA-seq reveals the cellular heterogeneity, developmental trajectories, and regulatory networks of immune cells across tissues. Analyses define T cell differentiation, thymic development, and microbiota-driven immune maturation, while disease-related studies uncover immune responses, host–pathogen interactions, and microenvironmental dynamics in infection, transplantation, and tumor contexts.

Recent advances in single-cell technologies have greatly expanded our understanding of the systemic organization, development dynamics, and pathological responses of the porcine immune system (Figure 5).

Early scRNA-seq studies of peripheral blood revealed pronounced heterogeneity among circulating CD8^+^ T cells, identifying distinct subpopulations, differentiation trajectories, and key regulatory transcription factors, thereby refining our understanding of porcine adaptive immunity and its translational relevance [72]. Extending these efforts to immune ontogeny, single-cell profiling of the early-adolescent pig thymus comprehensively mapped thymopoiesis, the process by which bone marrow progenitors migrate to the thymus and differentiate into mature T cells, and unconventional T cell subsets, demonstrating that porcine iNKT cell differentiation is dominated by iNKT2-like states and lacks classical mouse iNKT1/iNKT17 subsets, thus highlighting both conserved and species-specific features of T cell development [73].

Immune homeostasis has also been explored through microbiota-dependent regulation. Comparative single-cell transcriptomic analyses of germ-free and specific pathogen-free piglets revealed that microbial exposure profoundly shapes T, B, and myeloid cell composition and maturation across mesenteric lymph nodes, Peyer’s patches, and the spleen, underscoring the essential role of environmental cues in immune system establishment [74]. These findings primarily illuminate systemic immune organization.

In pathological contexts, single-cell approaches have provided mechanistic insights into infectious diseases, transplantation, and tumor microenvironments. In the African swine fever virus-infected spleen, single-cell analysis uncovered dynamic virus–host interactions, identifying macrophages and monocytes as primary viral targets with suppressed interferon responses, thereby illuminating mechanisms of immune evasion and disease progression [75]. Complementarily, single-cell antigen receptor sequencing in influenza-infected pigs characterized antigen-driven clonal expansion and diversification of T and B cell repertoires, revealing adaptive immune dynamics in a clinically relevant large-animal model [76]. Beyond infection, single-cell transcriptomic profiling of pig-to-human kidney xenotransplantation revealed coordinated immune activation, endothelial adaptation, and interspecies cell–cell interactions within the graft microenvironment, highlighting molecular pathways underlying xenograft rejection and accommodation [77]. Finally, in a porcine neurofibromatosis type 1 model, scRNA-seq of neurofibromas delineated a complex tumor microenvironment characterized by immune suppression, M2 macrophage enrichment, fibroblast-driven extracellular matrix remodeling, and transcriptional programs supporting neural regeneration and tumor progression [78].

Collectively, these studies establish a system-level framework for porcine immune organization, developmental plasticity, and disease responses. Single-cell analyses have identified cell-type-specific regulatory programs and host–pathogen interactions that provide candidate biomarkers for disease resistance, vaccine responsiveness, and immune robustness in breeding populations, enabling integration with genomic selection and precision livestock management. However, interpretation remains limited by technical variability, a lack of large-scale validation, and inconsistencies in cell-type annotation across studies. Moreover, while pigs serve as important translational models for human immunology and xenotransplantation, species-specific immune differences should be considered when extrapolating findings to human systems.

### 3.4. Porcine Growth and Metabolism

**Figure 6:**
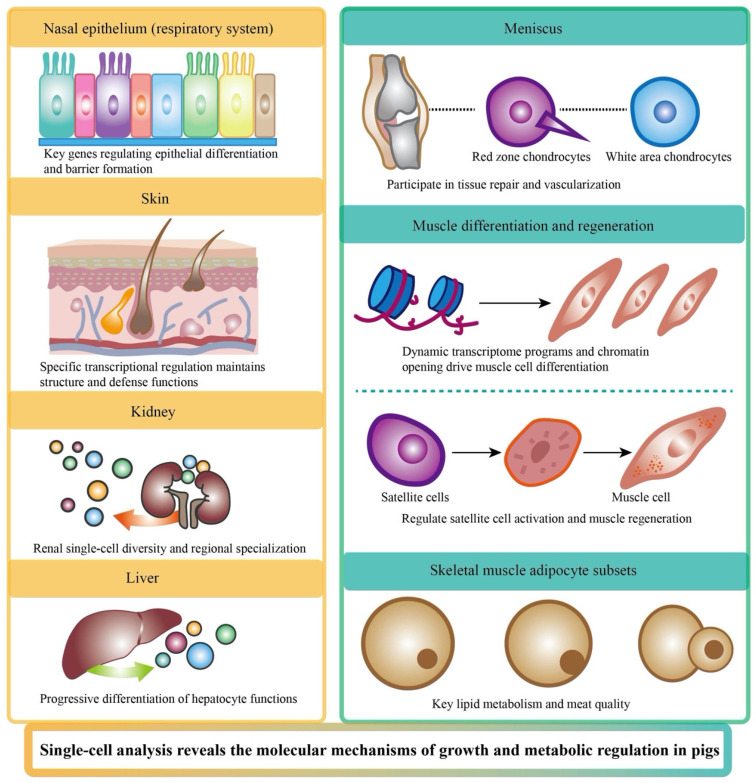
Single-cell atlas of porcine growth, tissue development, and metabolic specialization. scRNA-seq reveals cellular heterogeneity, developmental trajectories, and tissue-specific regulatory programs across multiple porcine organs. Analyses highlight epithelial differentiation, organ maturation, muscle development, adipocyte diversity, and metabolic adaptation, providing a comprehensive framework for understanding growth performance and tissue specialization in pigs.

Recent advances in single-cell sequencing have enabled the construction of comprehensive cellular atlases across diverse porcine tissues, providing a systematic framework for investigating tissue maturation, metabolic specialization, and growth-related regulatory programs (Figure 6). Methodological progress has supported these efforts, including the establishment of standardized protocols for isolating and cryopreserving pig tissues, such as the skin, while preserving cellular viability and transcriptional integrity, thereby facilitating reproducible large-scale single-cell analyses [79].

At the organ level, single-cell transcriptomics has revealed dynamic developmental trajectories and spatial heterogeneity underlying tissue maturation. In the respiratory system, profiling of the domestic pig nasal epithelium identified distinct basal, ciliated, and secretory cell populations, along with intermediate states and regulatory genes governing epithelial differentiation and barrier formation [80]. Similarly, a single-cell atlas of pig skin demonstrated pronounced anatomical positional heterogeneity, with keratinocyte, fibroblast, and immune cell subpopulations exhibiting location-specific transcriptional programs associated with structural maintenance and protective functions [81]. In metabolic and excretory organs, integrated single-cell maps of pig kidneys across developmental stages defined nephron cell diversity and regional specialization, uncovering the transcriptional programs associated with renal maturation [82]. Postnatal liver profiling further revealed progressive hepatocyte diversification and the coordinated establishment of metabolic and immune functions, reflecting functional adaptation during growth [83].

Single-cell approaches have been particularly informative for understanding musculoskeletal development and its implications for growth performance. In the pig meniscus, distinct cellular heterogeneity between red and white zones was identified, with red-zone chondrocytes enriched in stemness- and angiogenesis-related pathways, suggesting roles in tissue repair and vascularization [84]. Integrative single-cell RNA-seq and ATAC-seq analyses during myogenic differentiation delineated dynamic transcriptional and chromatin accessibility programs driving muscle lineage commitment [85]. Moreover, functional interactions between muscle satellite cells and fibro-adipogenic progenitors were characterized, demonstrating that FGF7-mediated signaling regulates satellite cell activation and myogenic differentiation during regeneration [86]. These findings collectively elucidate regulatory mechanisms underlying muscle growth and regenerative capacity.

Beyond muscle development, single-cell transcriptomic analyses of porcine skeletal muscle identified three distinct adipocyte populations with unique metabolic and transcriptional features, each strongly associated with intramuscular fat deposition [87]. These results provide cell-type-specific insights into lipid metabolism and meat quality regulation, directly linking cellular heterogeneity to economically important production traits.

Finally, cross-tissue single-cell analyses have revealed broader principles of cellular specialization and systemic regulation. A comprehensive pig cell landscape constructed at the single-cell resolution highlighted pronounced endothelial heterogeneity across tissues and distinct microglial regulatory networks, identifying tissue-specific endothelial subpopulations and conserved microglial regulons involved in vascular specialization and neuroimmune coordination [88]. Collectively, these studies show that single-cell approaches have shifted the understanding of porcine growth and metabolism from tissue-level descriptions to cell-type-resolved regulatory mechanisms underlying economically important traits. Key pathways involved in differentiation, metabolism, and signaling contribute to variation in growth performance, carcass traits, and meat quality, particularly through regulation of myogenesis and adipogenesis. However, current data remain fragmented across tissues and stages, limiting integrated interpretation of growth-regulatory networks. Future work should focus on cross-tissue integration and the incorporation of single-cell data into genomic selection to support precision breeding in pigs.

### 3.5. Porcine Intestinal Mucosal Ecosystem

**Figure 7:**
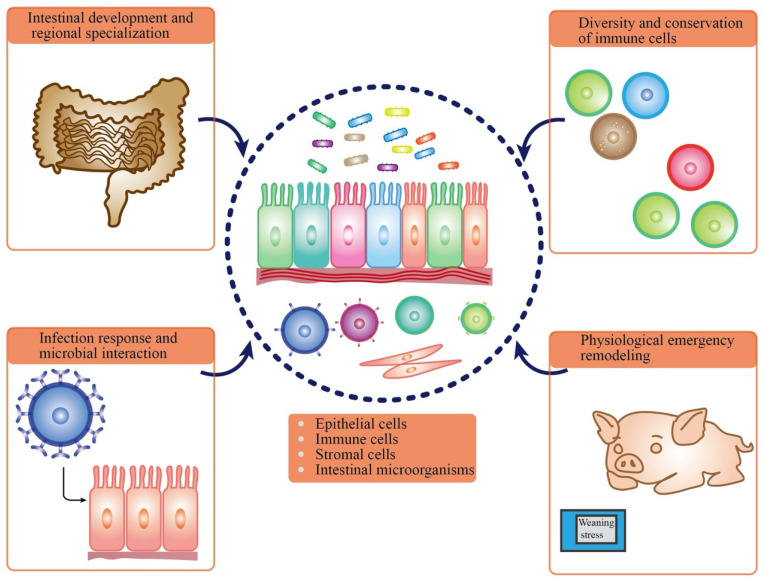
Single-cell landscape of the porcine intestinal mucosal ecosystem. scRNA-seq reveals the coordinated organization of epithelial, immune, and stromal cell populations and their interactions with the microbiota. Analyses highlight regional specialization along the intestinal tract, immune cell diversity, and dynamic responses to development, infection, and physiological stress, providing a comprehensive view of intestinal homeostasis and function in pigs.

Single-cell transcriptomic technologies have enabled systematic dissection of the porcine intestinal mucosal ecosystem, revealing the coordinated organization of epithelial, immune, and stromal compartments and their dynamic interactions with the microbiota (Figure 7). These studies provide high-resolution insights into intestinal development, regional specialization, immune homeostasis, and responses to infection and physiological stress. Single-cell analyses across developmental stages of the porcine cecum revealed dynamic remodeling of epithelial, immune, and stromal populations, with stage-specific transcriptional programs governing epithelial maturation, barrier formation, and immune establishment [89]. Complementary profiling of the small intestine further uncovered pronounced regional heterogeneity along the duodenum, jejunum, and ileum, with distinct epithelial subtypes displaying segment-specific transcriptional programs associated with nutrient absorption, metabolic regulation, and local immune modulation, reflecting functional specialization along the gut axis [35]. Together, these findings establish the structural and transcriptional basis of intestinal compartmentalization in pigs.

Beyond epithelial diversity, single-cell profiling has characterized diverse immune cell populations residing in the intestinal lamina propria and associated lymphoid structures. Novel lymphocyte subsets were identified, with some exhibiting transcriptional features conserved with human intestinal immune cells, thus supporting the pig as a valuable large-animal model for gut immunology [90]. Notably, innate lymphoid cell-like populations lacking TCR expression were detected in the jejunal lamina propria, highlighting conserved mechanisms of mucosal immune defense and epithelial–immune coordination [91]. Analyses of jejunal and ileal Peyer’s patches further revealed conserved B cell activation and differentiation programs despite regional immune differences, underscoring the stability of core humoral regulatory pathways across intestinal segments [92]. Single-cell approaches have also elucidated cellular responses to enteric viral infection. In a PEDV-infected small intestine, epithelial cells were identified as the primary viral targets, with cell-type-specific activation of antiviral-, inflammatory-, and barrier-related transcriptional programs [93]. Importantly, microbiota-derived factors modulate these responses. Outer membrane vesicles derived from *Faecalibacterium prausnitzii* alleviated PEDV infection by reshaping microbial metabolic pathways, enhancing epithelial barrier integrity, and dampening inflammation, demonstrating a microbiota-dependent mechanism of antiviral protection [94]. These findings highlight the central role of the epithelial–immune–microbiota axis in maintaining intestinal homeostasis under a pathogen challenge. Physiological stress such as weaning induces marked intestinal remodeling, characterized by altered epithelial and immune cell composition, impaired barrier function, and activation of inflammatory programs in the ileum, providing cellular insight into weaning-associated intestinal dysfunction [95]. At a broader scale, a comprehensive single-cell atlas comprising over one million porcine intestinal cells systematically mapped epithelial, immune, and stromal populations and their developmental trajectories, revealing conserved transcriptional programs across species and providing a foundational resource for comparative gut biology [96].

**Table 3:** Representative porcine scRNA-seq datasets discussed in this review.

System	Tissue	Platform	Main Findings	Accession	Reference
Embryonic development	Gastrulation	10× Genomics	Gastrula formation	GSE236766	[57]
	Embryos	Smart-seq2	Interspecies chimeras	GSE112380	[58]
	Embryos	Smart-seq2	Pluripotent stem cells	CRA003960	[59]
Reproduction	Testis	10× Genomics	Male germ cells	GSE174782	[62]
	Testis	10× Genomics	Male germ cells	GSE186479	[63]
	Testis	10× Genomics	Male germ cells	PRJNA1172176	[64]
Immunity system	Thymus	10× Genomics	Thymopoiesis and inkt cell maturation	GSE192520	[73]
	Peyer’s Patches, Mesenteric Lymph Node, and Spleen	10× Genomics	Commensal microbe–host immunity regulation	CRA018161	[74]
	Spleen	10× Genomics	African swine fever virus (ASFV)	PRJNA879060	[75]
Growth and metabolism	Skin	10× Genomics	Skin	GSE166561	[79]
	Nasal Mucosa	10× Genomics	Respiratory diseases	GSE274334	[80]
	Kidney	GEXSCOPE	Metabolism	PRJNA1218407	[82]
Intestinal mucosal ecosystem	Jejunal	10× Genomics	Intestinal immune function	GSE196388	[90]
	Jejunal	10× Genomics	Intestinal mucosal immunity	GSE232605	[92]
	Jejunal	10× Genomics	Intestinal pathophysiological and inflammatory process	GSE174112	[95]

Collectively, the porcine intestinal mucosa operates as an interconnected ecosystem where epithelial, immune, stromal, and microbial compartments jointly govern gut development, immunity, and nutrient utilization. These coordinated mechanisms directly underpin crucial production traits, including feed efficiency, gut resilience, disease resistance, and post-weaning adaptation. However, inconsistent sampling and fragmented host–microbiota datasets currently limit cross-study comparisons. Future multi-omics frameworks integrating single-cell transcriptomics, microbiome profiling, and functional validation will be essential in identifying regulatory targets for precision nutrition and sustainable pig production.

Cross-system integration reveals that porcine biological systems are fundamentally interconnected via shared molecular pathways. For instance, the Wnt/β-catenin pathway regulates both embryogenesis and tissue regeneration [57,97], while the PI3K-Akt-mTOR network links intestinal nutrient sensing to muscle protein synthesis, directly impacting feed efficiency [15,98]. Utilizing bioinformatic tools like CellChat and WGCNA for multi-tissue integration can uncover inter-organ cross-talk and systemic hub genes governing multi-trait phenotypes [99,100]. This integrated approach transcends isolated organ atlases, enabling the discovery of systemic biomarkers and multi-trait selection targets for swine breeding. Single-cell discoveries offer dual benefits for swine production and biomedicine. In agriculture, cell-type-specific markers can enhance genomic selection for production traits and disease resistance, enabling precision livestock farming. Furthermore, the pig is a vital model for human biomedicine and xenotransplantation [101]. However, transcriptomic divergence in immune and metabolic regulation necessitates rigorous cross-species validation. Future research must balance these strengths to ensure that single-cell insights drive both sustainable swine production and safe biomedical translation.

## 4. Future Perspectives in Pig Production

The rapid integration of single-cell technologies into porcine research is progressively bridging fundamental biology and practical livestock production. As demonstrated in studies of early embryogenesis and pluripotency transitions [55,56,57], cell-type-resolved analyses provide mechanistic insights into developmental competence and epigenetic regulation, offering opportunities to optimize in vitro fertilization, cloning efficiency, and early embryo survival. Similarly, single-cell profiling of testicular and oocyte development has identified stage-specific markers and regulatory networks governing gametogenesis [63,64,65], thus establishing molecular frameworks that may improve reproductive efficiency and germplasm quality evaluation. In the context of disease resistance, cell-resolved immune analyses have revealed heterogeneous T cell subsets, microbiota-dependent immune maturation, and virus–host interactions in ASFV and influenza infection models [72,73,74], highlighting potential cellular targets for enhancing immune resilience and vaccine responsiveness. Moreover, single-cell atlases of metabolic organs and skeletal muscle have uncovered cell-type-specific transcriptional programs associated with myogenesis, adipocyte heterogeneity, and intramuscular fat deposition [83,84,85], providing refined targets for improving growth performance and meat quality traits. Insights into intestinal epithelial–immune–microbiota interactions [93,94,95,96] further support precision strategies for mitigating weaning stress and enteric diseases through microbiota modulation and barrier function optimization.

In genetic improvement, conventional genomic selection (GS) primarily relies on static genome-wide SNP markers and often exhibits limited predictive accuracy for low-heritability traits. By enabling the identification of trait-relevant cellular subpopulations and cell-type-specific molecular signatures, scRNA-seq provides functional information beyond conventional genomic markers. Integrating these functional annotations into GS frameworks may facilitate the identification of causal variants and improve breeding value prediction for complex economic traits, including meat quality, disease resistance, and reproductive performance [10,102]. Specifically, future genomic prediction models are expected to evolve toward biology-driven frameworks through the integration of single-cell-derived functional information and the prioritization of genetic markers that are enriched in trait-relevant cell clusters. In reproductive and health management, scRNA-seq has generated high-resolution cellular atlases of spermatogenesis, folliculogenesis, embryonic development, and host–pathogen interactions, accelerating the discovery of biomarkers associated with fertility and immune resilience [103,104]. The translation of these cellular insights into routine herd management will require the development of affordable, cell-type-specific diagnostic biomarkers that facilitate the early screening of immune resilience and reproductive performance. Furthermore, by resolving tissue phenotypes at the cellular resolution, scRNA-seq offers new opportunities to improve feed efficiency, support precision livestock management, and promote a transition from population-based selection toward mechanism-driven genetic improvement in pigs.

Looking forward, the integration of single-cell transcriptomics with spatial profiling, epigenomic analyses, and large-scale genetic selection data is expected to facilitate cell-type-informed breeding strategies. To move beyond broad genetic correlations, a key priority is to integrate scRNA-seq with chromatin accessibility data (e.g., snATAC-seq) to finely map cell-type-specific regulatory elements and thereby identify the causal variants underlying complex traits. With the continuous advancement of technology, sequencing costs are gradually decreasing, which is expected to accelerate the transformation of single-cell discovery into precision breeding, health management, and sustainable pig production systems. Although the high cost of sequencing currently constrains large-scale industry screening, scRNA-seq can act as an upstream discovery pipeline to prioritize high-confidence functional markers for subsequent cost-effective integration into customized commercial SNP arrays. The establishment of a unified porcine cell ontology and standardized reference atlases will further enhance cross-study reproducibility and facilitate data interoperability across the porcine single-cell research community. While current single-cell atlases provide unprecedented cellular resolution, they lack spatial context. Therefore, spatial transcriptomics represents the next frontier for porcine “Organ Atlas” projects.

## 5. Conclusions

Single-cell RNA sequencing has revolutionized our understanding of porcine biology by providing a high-resolution view of cellular heterogeneity and dynamic processes across various tissues and developmental stages. This transformative technology has enabled the identification of distinct cell populations, lineage trajectories, and regulatory networks that were previously obscured in bulk transcriptomic analyses. In particular, scRNA-seq has expanded our knowledge of key biological processes, including embryogenesis, reproduction, immune function, growth regulation, and gut health, all of which are critical for improving pig production efficiency and disease management.

Despite the rapid advancements in single-cell technologies, challenges remain, including the need for cost-effective platforms, integration of multi-omics data, and standardization of protocols for large-scale studies. However, the increasing accessibility of scRNA-seq platforms and the growing availability of genomic resources are paving the way for more comprehensive and translational studies in pigs. Looking forward, the integration of single-cell transcriptomics with spatial technologies, functional genomics, and large-scale breeding programs holds great promise for advancing precision livestock farming. Furthermore, given the striking physiological and anatomical similarities between pigs and humans, porcine single-cell research is of paramount importance as it provides a critical translational bridge for biomedical applications. These advancements will likely lead to more sustainable, efficient, and resilient pig production systems, contributing to both agricultural productivity and biomedical applications.

## Figures and Tables

**Table 1:** Comparison of scRNA-seq platforms used in porcine studies.

Platform	Type	Throughput	Performance	Cost and Computational Demand	Advantages	Limitations	Pig Research Applications/References
Smart-seq2	Plate-based	Low	High capture efficiency; low doublet tendency; very high transcript sensitivity; deep sequencing	High cost; moderate computational demand	Full-length transcript coverage; high sensitivity; detection of low-abundance genes; allele/isoform resolution	Labor-intensive; low throughput; high cost per cell	Early embryos [13]; comparative platform evaluation [44]; Smart-seq2 protocol [43,45]
SMARTer ICELL8	Plate-based	Low	High capture efficiency; low doublet tendency; high transcript sensitivity; deep sequencing	High cost; moderate computational demand	High-resolution full-length sequencing; flexible cell selection; imaging-based QC	Low throughput; expensive reagents	Limited pig embryo studies; general workflow reference [30]
10× Genomics Chromium	Droplet-based	High	Moderate–high capture efficiency; moderate doublet tendency; moderate transcript sensitivity; moderate sequencing depth	Moderate cost; moderate computational demand	High throughput; cost-effective per cell; scalable; widely adopted; suitable for cell atlas projects	3′/5′ end bias; limited isoform detection	Pig testis atlas [14,35]; intestine [17,36]; immune cells [15]; skeletal muscle [16]
Drop-seq	Droplet-based	High	Moderate capture efficiency; moderate–high doublet tendency; low–moderate transcript sensitivity; moderate sequencing depth	Low cost; moderate computational demand	Low cost; scalable; early droplet innovation	Lower sensitivity vs. 10×; less standardized in pigs	Conceptual droplet foundation [48]; limited pig-specific reports
inDrop	Droplet-based	High	Moderate capture efficiency; moderate doublet tendency; moderate transcript sensitivity; moderate sequencing depth	Moderate cost; moderate computational demand	High-throughput droplet barcoding; early microfluidic platform	Less commercially supported; less used in recent pig studies	Methodological reference [49]; occasional immune/intestine applications
BD Rhapsody	Microwell/bead-based	Medium	High capture efficiency; low doublet tendency; high transcript sensitivity; moderate–high sequencing depth	Moderate–high cost; moderate computational demand	Higher sensitivity for low-input samples; targeted panels; immune profiling	Moderate throughput; proprietary consumables	Immune and blood cell analysis [52]
Singleron GEXSCOPE	Droplet-based	High	Moderate capture efficiency; high doublet tendency; moderate transcript sensitivity; moderate sequencing depth	Moderate cost; moderate computational demand	Cost-effective; scalable; increasing use in livestock	Limited international benchmarking	Emerging pig intestine/liver datasets (methodological reference [21,23])
CITE-seq/REAP-seq	Multimodal	Medium	Moderate capture efficiency; moderate doublet tendency; high transcript sensitivity; high sequencing depth	High cost; high computational demand	Simultaneous RNA + protein detection; resolves immune cell states	Higher cost; complex analysis	Immune heterogeneity studies (technology reference [38])
10× Multiome (RNA+ATAC)	Multimodal	Medium	Moderate capture efficiency; moderate doublet tendency; high transcript sensitivity; high sequencing depth	High cost; very high computational demand	Integrated transcriptome + chromatin accessibility; regulatory insights	Complex library preparation; bioinformatics intensive	Multi-omics livestock applications [20,37]; immune/muscle regulation

**Table 2:** Common analytical strategies and tools for scRNA-seq data analysis in pigs.

Analysis Step	Purpose	Commonly Used Tools/Methods	Representative Applications in Pigs	References
Quality control and filtering	Remove low-quality cells, doublets, and technical artifacts	Seurat; Scanpy; UMI-based filtering	QC of pig IVF embryos, testis atlases, and immune and intestinal datasets	[13,14,15,17,35]
Normalization and scaling	Correct for sequencing depth and technical variation	Log normalization (Seurat); SCTransform; UMI normalization	Cross-stage normalization in skeletal muscle and intestinal development studies	[16,17,36]
Dimensionality reduction	Visualize cellular heterogeneity	PCA; UMAP; t-SNE	Visualization of cell-type diversity in pig testes, immune cells, and embryonic datasets	[13,14,15,35]
Cell clustering	Identify transcriptionally distinct cell populations	Graph-based clustering (Seurat); Louvain/Leiden algorithms	Identification of germ cells, Sertoli cells, immune subsets, intestinal epithelial subtypes	[14,15,17,35,36]
Cell-type annotation	Assign biological identity to clusters	Marker gene analysis; cross-species comparison; reference-based annotation	Cross-species immune comparison (human vs. pig); reproductive and intestinal cell annotation	[15,35,36]
Trajectory inference/pseudotime analysis	Reconstruct dynamic biological processes	Monocle; pseudotemporal ordering	Spermatogenesis and puberty progression; embryonic lineage development; muscle differentiation	[13,16,33,35]
Cell–cell communication analysis	Infer ligand–receptor interactions and niche signaling	Ligand–receptor interaction analysis frameworks	Testis niche signaling; immune cell interactions; developmental signaling	[14,15,35]
Data integration and batch correction	Integrate datasets across conditions, stages, or species	Seurat integration; anchor-based integration; harmony-like strategies	Cross-stage and cross-species integration in porcine immune and reproductive studies	[15,31,32]
UMI-based quantification and barcode processing	Accurate molecule counting and duplicate removal	UMI strategies; BUStools	High-throughput droplet datasets in pig tissues	[27,28,29]
Multi-omics integration	Combine transcriptome with epigenome or proteome	RNA+ATAC integration; CITE-seq analysis	Livestock multi-omics integration; immune regulatory analysis	[20,37,38]

## Data Availability

No new data were created or analyzed in this study. Data sharing is not applicable to this article.
